# Superior Mesenteric Artery Syndrome: A Classic Presentation of a Rare Entity

**DOI:** 10.7759/cureus.9990

**Published:** 2020-08-24

**Authors:** Sunraj Tharu, Biswaraj Tharu, Mohammed Mahgoub, Muhammad Umar Khalid, Arooj Ahmed

**Affiliations:** 1 Audiology and Speech Language Pathology, Institute of Medicine (IOM), Kathmandu, NPL; 2 Internal Medicine, Western Reserve Health Education/Northeast Ohio Medical University (NEOMED), Warren, USA; 3 Medicine, Western Reserve Health Education/Northeast Ohio Medical University (NEOMED), Warren, USA; 4 Internal Medicine, Utica Medical Center, Warren, USA

**Keywords:** superior mesenteric artery, superior mesenteric artery syndrome, anorexia nervosa, rapid weight loss, sma

## Abstract

A 32-year-old female with a past medical history of constipation (predominant irritable bowel syndrome (IBS) and gastro-esophageal reflux disease (GERD)) presented with a complaint of pain in the lower abdomen. She lost 20 pounds in three months with a current body mass index (BMI) of 19.5 kg/m2 (ref: normal level 18.5-24.9). Computed tomography (CT) of the abdomen with contrast showed very little intra-abdominal fat, enlarged proximal duodenum, and decreased aorto-mesenteric angle of 15.4^0^ suggestive of superior mesenteric artery (SMA) syndrome. Per general surgery, the patient was managed conservatively: initially Nil Per Os (NPO), slowly transitioned to a clear liquid diet, soft diet, and solid diet. She tolerated the diet, improved clinically, and was discharged home.

## Introduction

Superior mesenteric artery (SMA) syndrome is a rare clinical entity with an incidence of 0.20% and 0.78% [1]. It occurs when the third part of the duodenum is compressed between the SMA and aorta. SMA syndrome is an unusual cause of proximal intestinal obstruction. It usually presents with non-specific symptoms as nausea, vomiting, pain abdomen. Sudden weight loss is its usual predisposing factor. Diagnosis can be challenging [2]. Treatment is done by conservative management approach and when it fails, surgical management can be indicated. We present a case of SMA syndrome in a 32-year-old female with a classic presentation and managed successfully with a conservative approach.

## Case presentation

A 32-year-old female with a past medical history of constipation (predominant irritable bowel syndrome (IBS) and gastro-esophageal reflux disease (GERD)) and surgical history of cholecystectomy, three caesarian sections (c-sections), hysterectomy, and bilateral tubal ligation for adenomyosis, five years back, presented with complain of pain in the lower abdomen. The pain had been there on/off for the last five years since her last c-section. She had associated nausea and vomiting.

**Figure 1:**
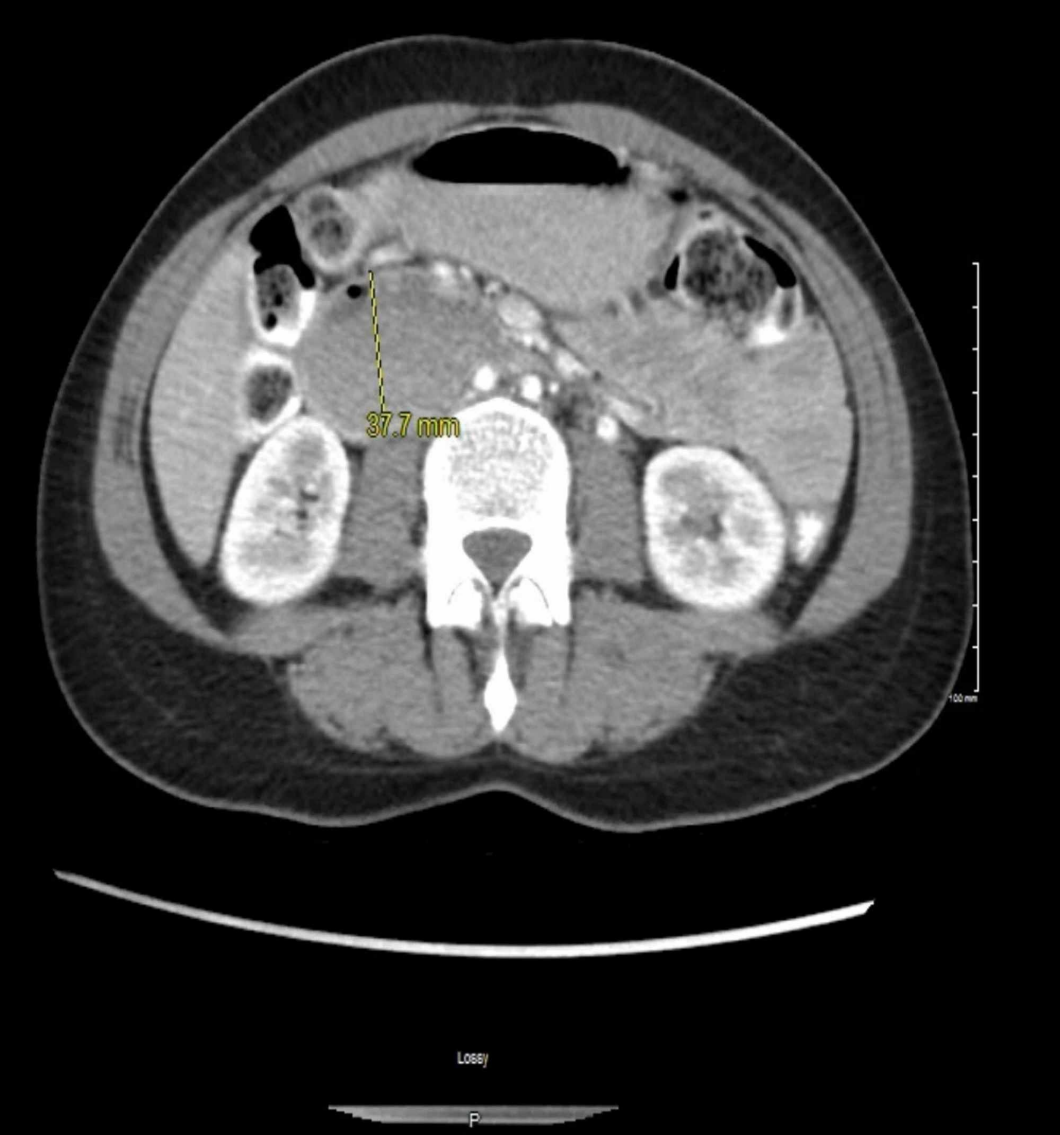
CT abdomen showing very little intra-abdominal fat, fluid-filled stomach, enlarged proximal duodenum measuring up to 37.7 mm

**Figure 2:**
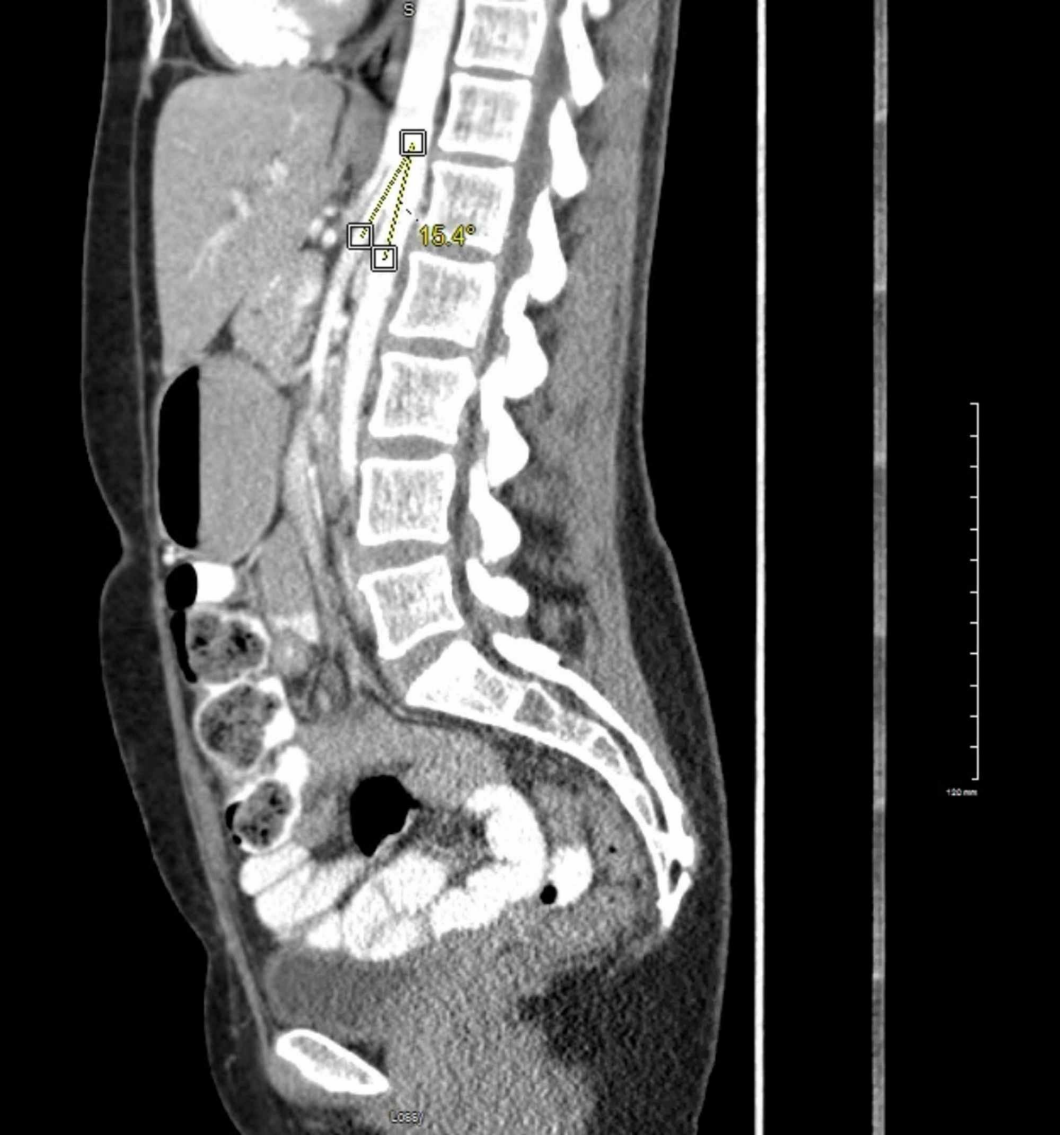
CT abdomen showing aorto-mesenteric angle of 15.4 degree

Her weight decreased 20 pounds in three months, from 114 pounds to the current weight of 94 pounds, which she attributed to lost appetite and inability to tolerate food with associated fullness and nausea. Her body mass index (BMI) was 19.5. Vitals were stable, no other significant exam findings were noted. Examination revealed non-tender, non-distended abdomen with bowel sounds positive in four quadrants and small < 1 cm palpable right inguinal lymph node which was non-tender. Computed tomography (CT) of the abdomen with contrast showed very little intra-abdominal fat, fluid-filled stomach, enlarged proximal duodenum measuring up to 37.7 mm in the anterio-posterior (AP) dimension with narrowing at the second portion and decreased aorto-mesenteric angle of 15.4^0^, suggestive of SMA syndrome (Figures 1-2).

General surgery was consulted who suggested conservative management and no need for any surgical intervention with a plan for upper gastrointestinal (GI) series. The patient was kept Nil Per Os (NPO) and on intravenous (IV) fluid.

AP X-ray of the abdomen taken prior to a planned upper GI series showed a relatively large amount of contrast (probably due to prior CT abdomen with contrast) within the colon so GI series was canceled. The patient was started on oral diet which she tolerated. She was improving clinically and was discharged home.

## Discussion

SMA syndrome (also known as Wilkie’s syndrome, chronic duodenal ileus, arteriomesenteric duodenal obstruction, or cast syndrome) occurs when the third portion of the duodenum is compressed between the SMA and the aorta. SMA syndrome is an unusual and rare cause of both acute and chronic high intestinal obstruction. The incidence rate is between 0.20% and 0.78% by radiographic study [1]. This syndrome can be challenging diagnostically [2].

The clinical presentation of SMA syndrome is variable and nonspecific, including nausea, vomiting, abdominal pain, and weight loss. The predisposing factors to develop SMA syndrome are rapid weight loss scenarios (e.g. anorexia nervosa, neoplastic, malabsorptive states, burns), head trauma (e.g. in cerebral palsy patient) and trauma/deformity of the spine (e.g. body cast, overextension). The fact that SMA syndrome almost never occurs in obese patients highlights the importance of nutritional states [3]. Significant weight loss is the most common factor for decreasing the aortomesenteric angle.

The diagnosis of SMA syndrome is based on clinical symptoms and radiologic evidence of obstruction. Imaging (abdominal X-ray, CT scans, upper GI series) can reveal decreased intra-abdominal and retroperitoneal fat, dilatation of the first and second part of the duodenum with an abrupt cutoff at the third part and decreased aortomesenteric angle and aorta-SMA distance [4]. An aortomesenteric artery angle is an angle between the aorta and the SMA. Normally the aortomesenteric angle is 25° to 60° and aortomesenteric distance is 10 to 28 mm. This is decreased to <25° and 2 to 8 mm respectively in the case of SMA syndrome [5]. An aortomesenteric angle of ≤25° is the most sensitive measure of SMA syndrome [6]. Retention of barium within the duodenum, characteristic vertical linear extrinsic pressure on the third portion of the duodenum, and duodenal dilation with or without gastric dilation in upper GI series are indicative of SMA syndrome [3].

Complications of SMA could be severe and may lead to dehydration, metabolic imbalance, and death. Gastric perforation and respiratory distress are also reported [7,8]. 

SMA syndrome is managed by conservative treatment approach. Patients might require nasogastric decompression. Fluid resuscitation, correction of electrolyte abnormalities with enteral/parenteral nutritional support for reversal of weight loss should be acted upon promptly. If the patient fails with conservative therapy, surgical intervention is indicated which includes mobilization of the ligament of Treitz, gastrojejunostomy, and duodenojejunostomy [9].

SMA syndromes have not been associated with obese patients [3]. However, there are no proper studies that show weight gain would prevent further episodes of SMA syndrome. One case report mentions that an SMA syndrome patient treated with weight gain was symptom-free after a one-year follow-up [10]. Even in our case, it has been six months already and the patient still is symptom-free.

## Conclusions

Rapid weight loss is a significant risk factor for SMA syndrome. It becomes important to keep this diagnosis as a differential in any young adult with a history of vomiting and weight loss. There is also psychological consideration like anorexia nervosa. General surgery, medicine, and psychiatry should be involved in multidisciplinary care. Although obese people are less associated with SMA syndrome, there lack good studies that show further prevention of future episodes of SMA syndrome. Nevertheless, the current management prompts towards nutritional replenishment of the patient. SMA syndrome is rare but has the potential to have dreaded complications like dehydration, metabolic imbalance, and death. This thus necessitates prompt diagnosis and management.
